# *Usp21* Knockout Causes Abnormal Lipid Metabolism in Mouse and Its Polymorphism Correlates with Hypercholesterolemia in Outpatients

**DOI:** 10.3390/ijms26199727

**Published:** 2025-10-06

**Authors:** Sailakshmi Iyer, Naoko Hattori, Hiroshi Okuda, Takeya Nakagawa, Satoshi Fujii, Takahiro Maeda, Haruhiko Koseki, Takashi Ito

**Affiliations:** 1 Department of Biochemistry, Nagasaki University Graduate School of Biomedical Sciences, Nagasaki 852-8523, Japan; 2 Department of Dermatology, Nagasaki University Graduate School of Biomedical Sciences, Nagasaki 852-8523, Japan; 3 Department of Bioscience and Bioinformatics, Kyushu Institute of Technology, Fukuoka 804-8550, Japan; 4 Department of Community Medicine, Nagasaki University Graduate School of Biomedical Sciences, Nagasaki 852-8523, Japan; 5 Department of Island and Community Medicine, Nagasaki University Graduate School of Biomedical Sciences, Nagasaki 852-8523, Japan; 6 Laboratory for Developmental Genetics, RIKEN Center for Integrative Medical Sciences, Yokohama 230-0045, Japan

**Keywords:** *Usp21*, *Fabp7*, *Nlrc5*, *Ppargc1a*, hypercholesterolemia

## Abstract

*Usp21*, a member of the ubiquitin protease family, plays a vital role in various biological functions. However, the effects of *Usp21* dysfunction remain incompletely understood. In this study, we generated *Usp21* knockout (KO) mice. Blood tests showed no impairment of liver function but did reveal elevated levels of total cholesterol (T-CHOL) and free fatty acid (FFA) in *Usp21* KO mice compared to wild-type (WT) mice. Next, we performed RNA-sequencing (RNA-seq) to identify genes that *Usp21* regulates. The results highlighted several candidate genes based on their biological relevance, and their expression levels were validated by RT-qPCR. The *Usp21* KO mice exhibited significant elevations in the expression of the genes *Fabp7*, *Nlrc5*, and *Ppargc1a,* which play an important role in lipid metabolism, compared to WT. These data suggest that *Usp21* may play roles in lipid metabolism in association with *Fabp7*, *Nlrc5* and *Ppargc1a*. To clarify the involvement of *USP21* in human hypercholesterolemia, we examined single-nucleotide polymorphisms (SNPs) around *USP21* in non-hypercholesterolemic and hypercholesterolemic outpatients. We found that the rs11421 SNP downstream of *USP21* was significantly associated with hypercholesterolemia. These data suggest that *Usp21* plays a role in mice and human lipid metabolism and that its polymorphism may be a diagnostic marker for human hypercholesterolemia.

## 1. Introduction

Ubiquitin ligases and deubiquitylases form a large family that catalyzes the bonding between the carboxy terminus of ubiquitin and lysine residues and the hydrolysis of the isopeptide bond at the amino terminus, respectively [1,2,3,4]. Among the deubiquitylase family, ubiquitin-specific peptidase 21 (*Usp21*), a cysteine protease, is highly conserved among species [5] and is known to play a role in intracellular processes and diseases such as bladder carcinoma [6] and pancreatic ductal adenocarcinoma [7]. Additionally, *Usp21* is known to be involved in epigenetic regulation as well [5]. Nakagawa et al. found that *Usp21* increases after partial hepatectomy and catalyzes the hydrolysis of nucleosome ubiquitylated H2A (ubH2A), which aids in the di- and trimethylation process of H3K4 and initiates transcription of several genes associated with liver regeneration. In addition, overexpression of *Usp21* in the liver has been shown to upregulate the *Serpina6* gene, which is downregulated during hepatocyte regeneration [5]. In addition to the full-length *USP21* consisting of 565 amino acids in humans and *Usp21* consisting of 566 amino acids in mice, Okuda et al. found a short variant of *Usp21* in mice. This variant is caused by alternative splicing of exon 2, resulting in the deletion of 87 amino acids from the amino terminus of the long isoform of *Usp21*. The *Usp21* short variant lacks nuclear export signal and thus localizes to the nucleus better than the *Usp21* long variant [8]. Despite these findings, the impact of *Usp21* dysfunction on disease remains to be elucidated.

Hypercholesterolemia, also referred to as dyslipidemia, is a result of elevated levels of cholesterol in the blood. One of the genetic factors is the low-density lipoprotein (LDL) receptor (*LDLR*), which is involved in lipid metabolism and whose mutations are implicated in the development of atherosclerotic cardiovascular diseases (ASCVD) [9,10]. However, such mutations are relatively rare and do not account for the majority of hypercholesterolemia cases.

In this report, we generated *Usp21* KO mice and identified genes regulated by *Usp21*. Since hepatocyte regeneration after hepatectomy upregulated the *Usp21* gene, we expected that *Usp21* KO affects genes related to hepatocyte regeneration. We observed that the genes *Fabp7*, *Nlrc5*, and *Ppargc1a,* which are related to lipid metabolism, showed significant elevation in *Usp21* KO mice compared to WT mice. Moreover, blood tests revealed that *Usp21* KO mice exhibited elevated T-CHOL, and FFA levels as phenotype. In humans, we examined SNPs around *USP21* in non-hypercholesterolemic and hypercholesterolemic outpatients and found an association between hypercholesterolemia and the rs11421 SNP downstream of *USP21*. These data suggest that *Usp21* plays a role in lipid metabolism and that its polymorphism could serve as a pre-diagnostic marker for hypercholesterolemia.

## 2. Results

### 2.1. Generation and Validation of Usp21 KO Mice

**Figure 1:**
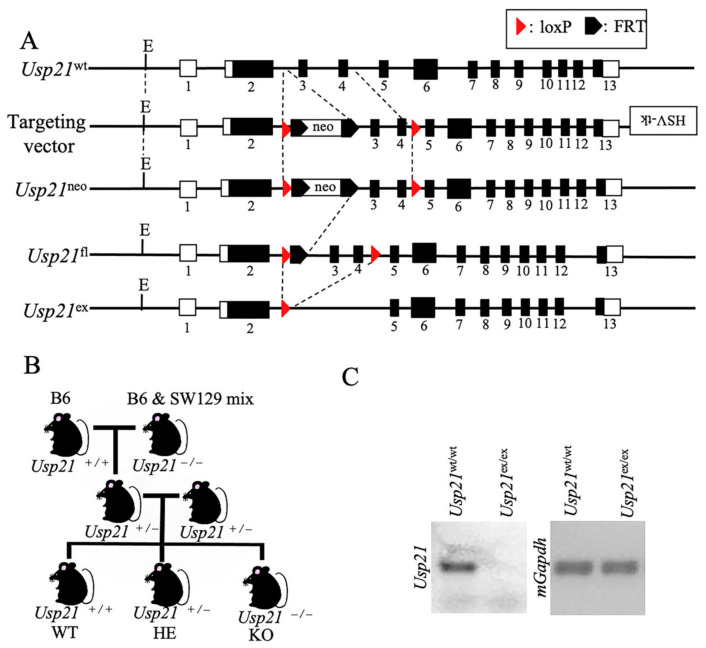
Generation and genotyping of *Usp21* KO mice. (**A**) Schematic representation of the *Usp21* locus and targeting strategy. Line 1, *Usp21* wild-type (WT) allele; line 2, targeting vector containing an HSV–thymidine kinase (HSV-tk) cassette, an FRT-flanked neomycin resistance cassette, and loxP sites were inserted at two positions as indicated; line 3, *Usp21*^neo^ allele following homologous recombination; line 4, *Usp21*^fl^ allele after removal of the neo cassette by Flp recombinase; line 5, *Usp21*^ex^ allele after Cre-mediated excision of exons 3 and 4. Dotted lines indicate regions targeted for recombination. Black boxes represent exons, and white boxes represent untranslated regions (UTRs). Numbers denote exon numbers. (**B**) Breeding strategy for generating *Usp21* KO mice. C57BL/6 (B6) mice carrying the WT allele were crossed with B6 × 129/Sv mixed-background mice harboring the *Usp21* KO allele to obtain littermates of different genotypes. (**C**) Genotyping of *Usp21* mice by PCR. **Right**, PCR amplification with primers spanning the loxP and recombined loci distinguishes *Usp21*^wt/wt^ and *Usp21*^ex/ex^ alleles. **Left**, PCR amplification of *mGapdh* served as an internal control.

To investigate the biological role of *Usp21*, we generated KO mice by targeted deletion of critical coding exons. The targeting vector contained an FRT-flanked neomycin resistance cassette and loxP sites positioned to flank exons 2–5, which include exons 3 and 4 that encode essential residues of the USP catalytic domain [11] (Figure 1A). Following homologous recombination in 129/Sv-derived ES cells, correctly targeted ES cell clones were injected into C57BL/6 blastocysts, which then generate chimeric mice. Heterozygous offspring carrying the targeted allele were identified by PCR genotyping. These mice were then crossed with Flp recombinase-expressing transgenic mice to remove the FRT-flanked neo cassette, thereby minimizing potential interference of the selection marker with gene expression. The resultant allele retained loxP sites at two positions: one between exons 2 and 3, and the other between exons 4 and 5. Subsequently, the floxed mice were bred with Cre recombinase-expressing mice to excise exons 3 and 4 in vivo. Exons 3 and 4 are an 181bp long sequence, and the stop codon appeared immediately after entering exon 5. This strategy yielded constitutive KO rather than conditional KO mice, because deletion of the critical exons abolished the gene function in all tissues.

To generate experimental mouse, heterozygous (HE) mice were intercrossed with B6 mice. Littermates were obtained by breeding B6 mice carrying the *Usp21* WT alleles with B6 × 129/Sv mixed-background mice harboring the KO alleles, thereby yielding WT and KO offspring for downstream analyses (Figure 1B). PCR genotyping was confirmed by PCR with primers spanning the loxP and recombined loci distinguished WT and KO alleles, as shown by DNA gel electrophoresis (Figure 1C).

### 2.2. Usp21 KO Mice Showed an Elevation in Serum FFA and T-CHOL

**Figure 2:**
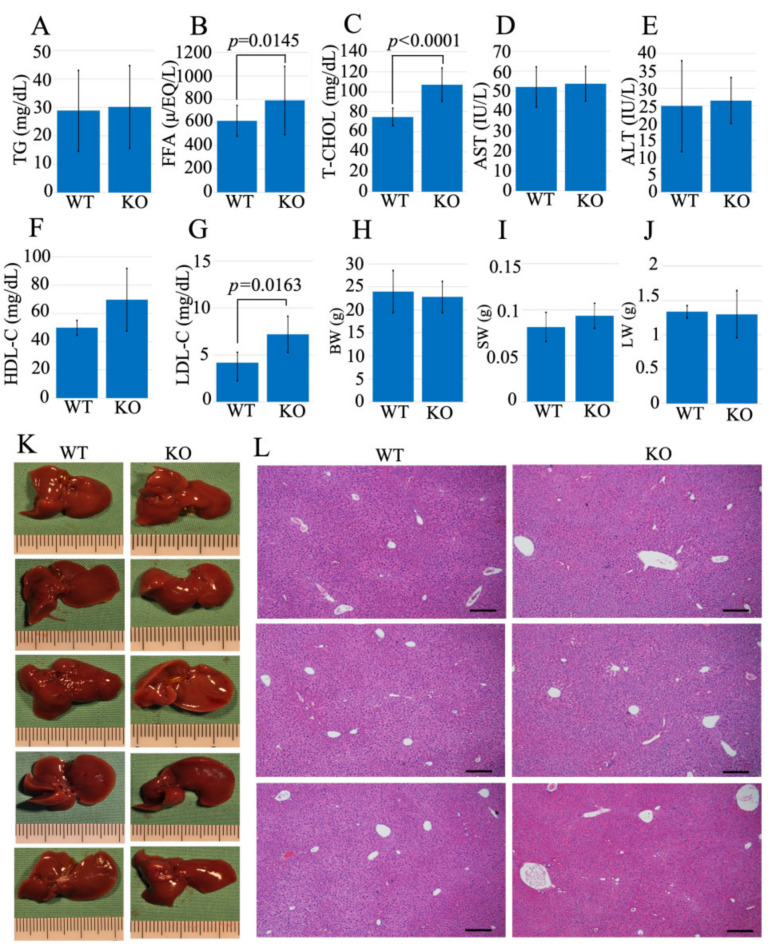
FFA and T-CHOL were elevated in *Usp21* KO mice. (**A**–**G**) The blood test data of (**A**) TG, (**B**) FFA, (**C**) T-CHOL, (**D**) AST, (**E**) ALT, (**F**) HDL-C, (**G**) LDL-C, (**H**) BW, (**I**) SW, (**J**) LW, (**K**) liver images, and (**L**) HE staining of WT, and *Usp21* KO mice. Scale bar 100 μm. All mice used in these experiments were 10–11 weeks old. WT (*n* = 21), and *Usp21* KO mice (*n* = 28). (Sample sizes vary by panel, Table S2). ALT, alanine transaminase; AST, aspartate aminotransferase; BW, body weight; FFA, free fatty acid; HDL-C, high-density lipoprotein cholesterol; H&E, hematoxylin and eosin; KO, knockout; LW, liver weight; LDL-*C*, low-density lipoprotein cholesterol; SW, spleen weight; TG, triglyceride; T-CHOL, total cholesterol; WT, wild-type. Student’s *t*-test was used to determine the statistical significance between the mice genotypes and blood test parameters (**A**–**J**). *p* < 0.05 is considered significant.

A blood test determined triglyceride (TG), free fatty acid (FFA), aspartate aminotransferase (AST), alanine transaminase (ALT), total cholesterol (T-CHOL), high-density lipoprotein cholesterol (HDL-C), and low-density lipoprotein cholesterol (LDL-C) serum levels in the littermate of 10–11 weeks WT, and *Usp21* KO mice (Figure 2A–G). The body weight (BW), spleen weight (SW), and liver weight (LW) were measured simultaneously (Figure 2H–J). The results showed no significant changes in the liver enzymes AST and ALT, confirming that liver function was not impaired. However, the levels of FFA and T-CHOL were significantly elevated in *Usp21* KO mice compared to WT with *p*-values (*p* = 0.0145) and (*p* < 0.0001), respectively. The levels of LDL-C and HDL-C indicated an increase in *Usp21* KO mice compared to WT controls, (Figure 2F,G) with LDL-C being significant with a *p*-value of *p* = 0.0163. There was no difference in the macroscopic appearance of the liver between the WT and *Usp21* KO groups (Figure 2K). Hematoxylin and eosin (H&E) staining of liver tissue from both WT and *Usp21 KO* mice (Figure 2L) further confirmed a normal microscopic appearance in *Usp21* KO mice, with no evidence of fatty liver.

Elevated FFA levels have been linked to metabolic and cardiovascular diseases [12,13], and the observed increase in FFAs in *Usp21* KO mice suggests that this model may be relevant for studying atherosclerosis, particularly in the context of elevated FFA. *Usp21* KO mice also exhibit increased levels of LDL-C, consistent with previous reports showing that elevated LDL-C is associated with cardiovascular disease in humans [14,15,16]. However, it is important to note that HDL in mice encompasses both HDL and LDL fractions found in humans [17]. Therefore, the elevated LDL-C and T-CHOL levels observed in *Usp21* KO mice compared to WT suggest a potential susceptibility to atherosclerosis in this model.

### 2.3. RNA-Seq Identifies Lipid Metabolism–Associated Genes Fabp7, Nlrc5, and Ppargc1a in Usp21 KO Mice

**Figure 3:**
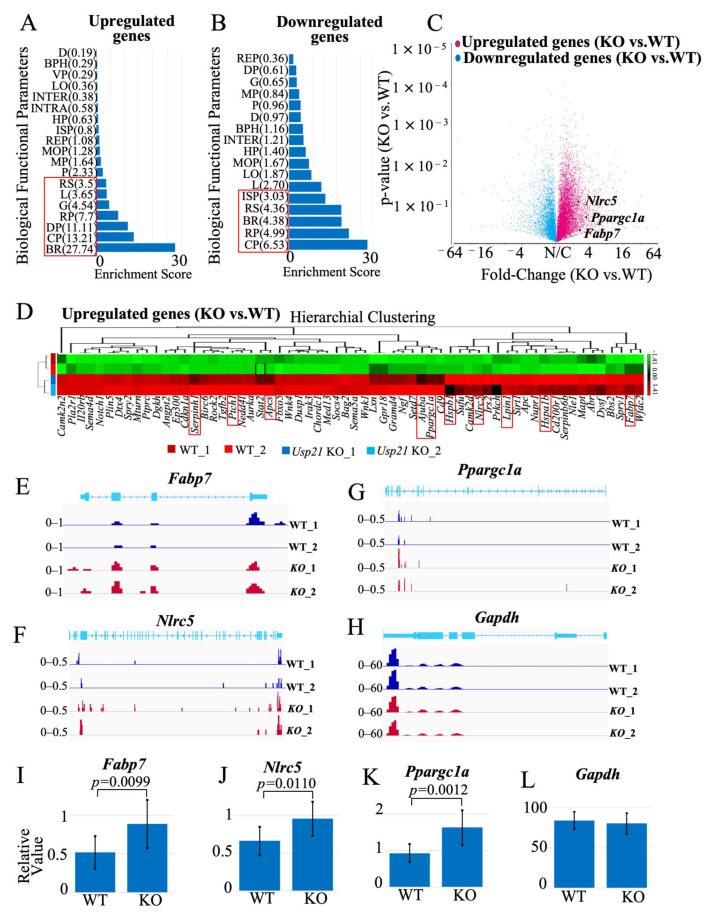
*Fabp7*, *Nlrc5,* and *Ppargc1a* are upregulated in *Usp21* KO mice. (**A**,**B**) GO analysis of (**A**) up-regulated genes and (**B**) down-regulated genes in *Usp21* KO mice, respectively. The red box represents a functional group with an enrichment score greater than 3, corresponding to an over-representation with *p* < 0.05. (**C**) Volcano plot depicting the 3 up-regulated genes (*Fabp7*, *Nlrc5*, and *Ppargc1a*) in WT and *Usp21* KO mice that were chosen for this study. (**D**) Heatmap of up-regulated genes in *Usp21* KO mice compared to WT mice. The top 10 up-regulated genes are highlighted with a red box. (**E**–**G**) Chromosome view of upregulated genes *Fabp7*, *Nlrc5*, and *Ppargc1a* in *Usp21* KO mice in comparison with WT mice. (**H**) Chromosome view of the reference gene *Gapdh*. (**I**–**K**) RT-qPCR of *Fabp7*, *Nlrc5*, and *Ppargc1a*, respectively. The values are normalized with the *Gapdh* expression (**L**). BPH, Biological Phase; BR, Biological regulation; BP, Biological phase; CP, Cellular process; D, Detoxification; DP, Developmental process; G, Growth; HP, Homeostatic process; INTER, Biological process involved in interspecies interaction between organisms; INTRA, Biological process involved in intraspecies interaction between organisms; ISP, Immune system process; KO, knockout. L, Localization; LO, Locomotion; MOP, Multicellular organismal process; MP, Metabolic process; P, Pigmentation; R, Reproduction; REP, Reproductive process; RP, Rhythmic process; RS, Response to stimulus; VP, Viral process; WT, wild type. Data are shown as mean ± SD. *p* values were tested with a student *t*-test. *p* < 0.05 is considered significant.

To identify genes regulated by *Usp21* in the liver, we performed RNA-seq analysis of liver tissues from WT and *Usp21* KO mice. Differential expression analysis revealed a substantial number of transcripts altered by at least 2-fold in *Usp21* KO livers and were analyzed using ANOVA in Partek Genomics Suite, followed by Gene Ontology (GO) analysis (Figure 3A, B). A functional group with an enrichment score of more than three corresponds to an over-representation with a *p* < 0.05. Among these, several genes implicated in lipid metabolism were significantly upregulated. Top 10 upregulated and downregulated genes were shortlisted based on their involvement in lipid metabolism. We prioritized genes based on functional relevance to lipid homeostasis, given that *Usp21* KO mice displayed elevated levels of FFA, T-CHOL, and LDL-C. Among the upregulated genes, *Fabp7*, *Nlrc5*, and *Ppargc1a* were selected for further analysis due to their known roles in fatty acid transport, immune-lipid crosstalk, and mitochondrial lipid oxidation, respectively. A volcano plot highlighting these genes is shown in (Figure 3C), and their expression profiles are visualized in the heatmap (Figure 3D) and genome browser views (Figure 3E–G). *Gapdh* expression was unaltered and served as a normalization control (Figure 3H).

The top 10 upregulated genes that were considered for this study included ajuba LIM protein (*Ajuba*), amyloid P component, serum (*Apcs*), fatty acid binding protein 7 (*Fabp7*)*,* heat shock protein 1B (*Hspa1b*), heat shock protein 1 (*Hspb1*), lipin 1 (*Lpin1*), NLR family CARD domain containing 5 (*Nlrc5*), peroxisome proliferator activated receptor gamma coactivator 1 alpha (*Ppargc1a*), patched 1 (*Ptch1*), and serine (or cysteine) peptidase inhibitor, clade H, member 1 (*Serpinh1*), (Figure 3D, Supplementary Figure S1). Conversely, the top 10 downregulated genes were: C-C motif chemokine ligand 2 (*Ccl2*), C-C motif chemokine receptor 7 (*Ccr7*), cytochrome P450, family 2, subfamily a, polypeptide 5 (*Cyp2a5*), Fanconi anemia core complex associated protein 100 (*Faap100*), G protein subunit alpha transducin 1 (*Gnat1*), KIT proto-oncogene receptor tyrosine kinase (*Kit*), NAD(P)H dehydrogenase, quinone 1 (*Nqo1*), proprotein convertase subtilisin/kexin type 9 (*Pcsk9*), retinoid X receptor gamma (*Rxrg*), Toll-like receptor 12 (*Tlr12*) with their heatmaps and chromosome view depicted in Supplementary Figure S2.

RT-qPCR confirmed the RNA-seq findings: *Fabp7*, *Nlrc5*, and *Ppargc1a* were significantly upregulated in *Usp21* KO livers compared to WT (*p* < 0.0099, *p* = 0.0110, and *p* = 0.0012, respectively; Figure 3I–K), normalized to *Gapdh* (Figure 3L).

Fatty acid binding protein 7 (*Fabp7*), a member of the intracellular lipid-binding protein family, facilitates fatty acid uptake, oxidation, and lipolysis. While primarily expressed in the brain, *Fabp7* is also present in hepatic Kupffer cells and has been implicated in regulating lipid profiles in peripheral tissues, such as skeletal muscle and liver [18,19,20,21,22,23,24].

*Nlrc5* is a member of the NOD-like receptor (NLR) family involved in immune regulation and metabolic homeostasis. *NLRC5* has been associated with plasma lipid traits, including TG, T-CHOL, and HDL-C levels [25,26,27,28]. *Nlrc5* deficiency in mice exacerbates obesity-related phenotypes under high-fat diet conditions [29].

*Ppargc1a* encodes PGC-1α, a transcriptional coactivator critical for mitochondrial fatty acid oxidation and adaptive energy metabolism. In hepatocytes, PGC-1α promotes lipid catabolism and downregulates TG secretion during fasting. It also upregulates genes involved in the tricarboxylic acid cycle and peroxisomal β-oxidation [30,31,32,33,34].

Together, these results suggest that loss of *Usp21* activates transcriptional programs involved in lipid handling through upregulation of *Fabp7*, *Nlrc5*, and *Ppargc1a*. Although GO analysis (Figure 3A,B) did not directly highlight lipid metabolism, the gene-level data strongly support a role for *USP21* in hepatic lipid regulation.

### 2.4. rs11421 SNP Is Associated with Hypercholesterolemia

**Figure 4:**
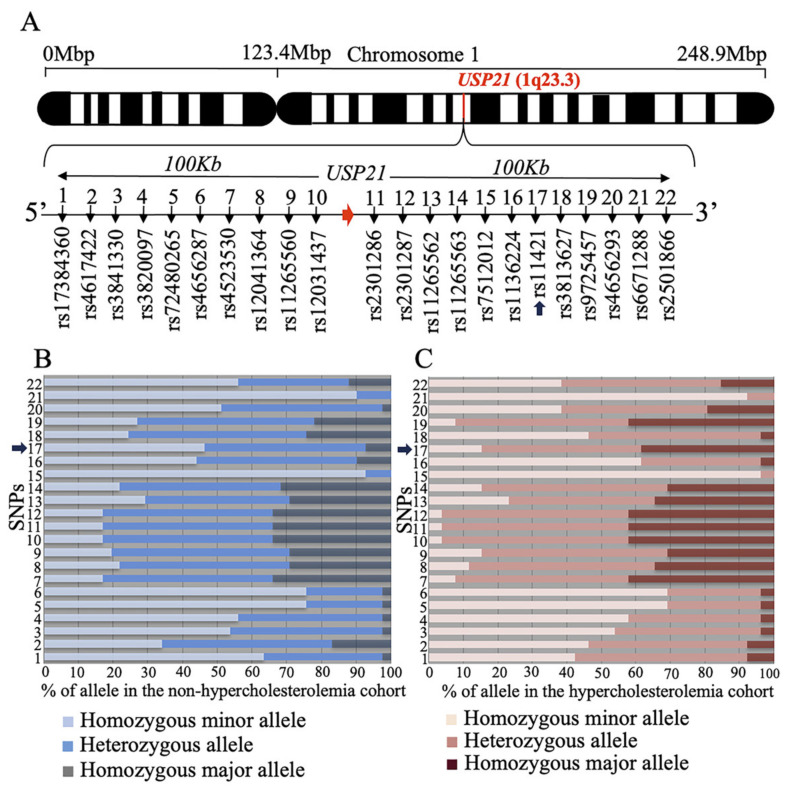
rs11421 is associated with hypercholesterolemia. (**A**) Schema of SNPs surrounding the human *USP21* gene. (**B**) Genotypes at each SNP locus in non-hypercholesterolemic outpatients. (**C**) Genotypes at each SNP locus in hypercholesterolemic outpatients. SNP, single-nucleotide polymorphism. Arrows indicate rs11421 SNP (**A**–**C**).

To investigate whether the human *USP21* gene is involved in hypercholesterolemia, we selected SNPs around the *USP21* gene based on GRCh37 and conducted an analysis (Figure 4A). After selecting the SNPs, we screened a sample of patients consisting of low LDL-C (<50 mg/dL) and high LDL-C (>160 mg/dL) for significant SNPs associated with serum cholesterol levels and performed a chi-squared test to determine statistical significance. We found that out of the 22 SNPs, the rs11421 SNP (Xsp I site ctag/ccag) showed statistical significance (Figure 4B, C). We observed a higher frequency of the ccag allele in high-LDL-C patients compared to low-LDL-C patients (*p* = 0.0093). Therefore, the CCAG allele of SNP rs11421 is significantly associated with hypercholesterolemia. This SNP, which potentially contributes to hypercholesterolemia, is located in the 3′ UTR of the *FCER1G* (Fc fragment of IgE receptor Ig) gene, approximately 53.8 kb away from *USP21,* depicted in Supplementary Figure S3.

The *FCER1G* gene encodes the γ chain of the Fc receptor and is probably unrelated to hypercholesterolemia. However, the simplicity of PCR amplification of SNP rs11421 followed by Xsp1 restriction enzyme cleavage contributes to predicting hypercholesterolemia in pre-diagnostic patients.

## 3. Discussion

We previously demonstrated that *Usp21* expression is induced during hepatocyte regeneration and that *Usp21* promotes transcriptional activation by deubiquitylating ubH2A. In vitro, *Usp21* directly removed ubiquitin from ubH2A, supporting its role in transcriptional regulation. Based on these findings, we hypothesized that *Usp21* KO would disrupt gene regulation during liver regeneration. However, *Usp21* KO mice displayed no overt defects in regeneration, with the only consistent phenotype being dysregulated lipid metabolism. To explore this further, we conducted transcriptomic profiling of liver tissue from WT, and *Usp21* KO mice. This analysis revealed upregulation of several lipid metabolism related genes, including *Fabp7*, *Nlrc5*, and *Ppargc1a*, which may account for the observed increases in serum FFA, T-CHOL, and LDL-C.

*Fabp7*, a lipid-binding protein traditionally associated with the brain, functions as a chaperone for intracellular fatty acids, mediating lipid uptake, transport, storage, and signal transduction. Its upregulation in *Usp21* KO liver implicates a previously unrecognized role in hepatic lipid homeostasis. Similarly, *Nlrc5*, previously linked to circulating HDL-C levels in humans [35] has been associated with various metabolic traits. Methylation at the *NLRC5* locus correlates with obesity-related parameters, such as body mass index and waist circumference [36], while hypomethylation has been reported in obese children compared to controls [26]. *Ppargc1a*, which encodes the transcriptional coactivator PGC-1α, is a master regulator of mitochondrial biogenesis and fatty acid oxidation [30], and its upregulation is consistent with compensatory responses to metabolic stress.

Several studies further support the involvement of these genes in lipid dysregulation. *Fabp7* is induced in HFD-fed mice, and its downregulation via miR-21 contributes to the protective effects of dietary lycopene against hepatic steatosis [37]. *Nlrc5* is best characterized as an immune-related transcriptional regulator that modulates MHC class I expression and innate immune responses, rather than as a classical metabolic enzyme [38,39]. However, some studies have reported that *Nlrc5* is implicated in liver fibrosis [40] and hepatocellular carcinoma [41], and that its expression is elevated in mouse livers following ethanol exposure, accompanied by increased T-CHOL, TG, ALT, and AST [42]. These findings suggest that altered expression of *Nlrc5* may, directly or indirectly, influence lipid metabolism in addition to its established role in immune activation pathways. PGC-1α is highly expressed in metabolically active tissues including the heart, brown adipose tissue, and kidney. Mice lacking *Ppargc1a* show impaired mitochondrial gene expression and develop early onset cardiac dysfunction due to defective oxidative phosphorylation and fatty acid oxidation [43]. Together, the coordinated upregulation of *Fabp7*, *Nlrc5*, and *Ppargc1a* in *Usp21* KO livers suggests a functional link between *Usp21* and hepatic lipid metabolism. Future loss-of function studies targeting these genes individually or in combination will be essential to clarify their contributions to the hyperlipidemic phenotype observed in *Usp21* deficient mice.

To further clarify the underlying molecular mechanisms beyond *Fabp7, Ppargc1a*, and *Nlrc5*, we analyzed representative genes involved in major lipid metabolic pathways using RNA-seq data. First, we examined lipoprotein-related genes. Apolipoprotein B (*Apob)* encodes the structural backbone of VLDL and LDL particles, and its secretion requires lipidation mediated by microsomal triglyceride transfer protein (*Mttp)* [44]. Apolipoprotein E (*Apoe)* mediates hepatic clearance of triglyceride rich lipoproteins, and its deficiency leads to severe hypercholesterolemia and atherosclerosis [45]. Apolipoprotein A-1 (*Apoa1)* is the major component of HDL, promoting reverse cholesterol transport and exerting protective effects against cholesterol accumulation and inflammation [46]. However, no significant changes in the expression of these genes were detected in *Usp21* KO. Next, we evaluated key enzymes of de novo fatty acid synthesis. ATP citrate lyase (*Acly)* provides cytosolic acetyl-CoA, acetyl-Coenzyme A carboxylase alpha (*Acaca)/* acetyl-Coenzyme A carboxylase beta (*Acacb)* catalyze the rate limiting conversion of acetyl-CoA to malonyl-CoA, and fatty acid synthase (*Fasn)* synthesizes palmitate [47]. Again, no clear alterations in the expression of these genes were observed in *Usp21* KO livers. Taken together, these findings indicate that the lipid abnormalities associated with *Usp21* deficiency are not attributable to transcriptional dysregulation of classical apolipoproteins or fatty acid synthesis enzymes but are more likely linked to the upregulation of *Fabp7*, *Ppargc1a*, and *Nlrc5*.

Interestingly, despite being maintained on a normal diet, *Usp21* KO mice exhibited significantly elevated T-CHOL and LDL-C levels. While mice are typically resistant to atherosclerosis due to their low basal LDL-C and predominant HDL mediated cholesterol transport, experimental models using HFD or *Apoe* and *Ldlr* KO have successfully induced atherosclerotic lesions [17,48,49,50,51,52,53]. In humans, HDL primarily functions as a cholesterol scavenger, whereas in mice, HDL also serves as the major plasma cholesterol carrier, an interspecies difference that partly explains their atheroresistance. Nonetheless, T-CHOL remains a critical determinant of atherogenic risk in both species.

The increase in plasma total cholesterol and LDL-C levels observed in *Usp21 KO* mice (~1.4-fold and ~1.7 fold compared with wild-type, respectively) is modest compared with the marked elevations in classical hypercholesterolemic models such as *Apoe*^−^ or *Ldlr*^−^ deficient mice, which develop spontaneous atherosclerosis [54]. Nevertheless, even a moderate increase in T-CHOL and LDL-C has been shown to contribute to cardiovascular risk in humans, where a 1.2–1.3-fold elevation is considered significant for the development of atherosclerosis over time [55]. Thus, although *Usp21* KO mice do not display the extreme lipid elevations in *Apoe* or *Ldlr* knockouts, the phenotype is valuable for studying milder dyslipidemia, which is highly prevalent in patients and clinically important as a risk factor for arteriosclerosis. This suggests that *Usp21* deficiency may contribute to lipid imbalance and cardiovascular risk through mechanisms distinct from those in established hypercholesterolemia models.

The spontaneous hypercholesterolemia observed in *Usp21* KO mice indicates a disruption of systemic cholesterol homeostasis and may predispose these animals to atherosclerosis with age or dietary challenge. Thus, *Usp21* KO mice provide a useful model for investigating the molecular basis of cholesterol metabolism and its pathological consequences. Importantly, combining *Usp21* deficiency with *Apoe* or *Ldlr* knockouts may further modify atherogenic phenotypes. While *Usp21* loss could exacerbate dyslipidemia in these hypercholesterolemic backgrounds, it is also possible that the severe lipid elevations inherent to these models would mask its effects. Future studies using such double-mutant mice will be critical to define the context dependent role of *Usp21* in lipid homeostasis and atherosclerosis risk.

Although we did not assess aortic lipid accumulation or plasma inflammatory markers in this study, future work addressing these parameters will be important to determine whether the moderate hypercholesterolemia in *Usp21* KO mice leads to early atherosclerotic changes and inflammatory responses associated with cardiovascular disease.

ASCVD remains the leading global cause of morbidity and mortality, with elevated LDL-C being a key risk factor. Hypercholesterolemia can arise from both genetic and environmental causes. The most common inherited form, familial hypercholesterolemia, results from mutations in the *LDLR* gene, with over 2200 unique variants documented in the University College London database [56,57,58,59]. However, the most prevalent form today is acquired hypercholesterolemia, driven by sedentary lifestyles and excessive intake of high-fat, low-fiber diets rich in trans fats. In this context, prevention through lifestyle modification including reduced dietary fat and increased fiber intake has become more critical than pharmacologic intervention alone [56]. In parallel with our mouse studies, we identified a significant association between the *USP21*-linked SNP rs11421 and hypercholesterolemia in a human cohort. Although it remains unclear whether *USP21* dysregulation directly drives hypercholesterolemia in humans, this variant may be associated with cholesterol imbalance. Importantly, large-scale and consistent studies will be needed to validate this relationship before rs11421 can be considered a predictive marker. Nonetheless, this clinical association supports the relevance of our mouse findings and highlights *USP21* as a potential regulator of lipid homeostasis and cardiovascular risk.

## 4. Materials and Methods

### 4.1. Generation of Usp21 KO Mice by Cre–LoxP System

A targeting vector was constructed with an FRT-flanked neomycin resistance cassette inserted between exons 2 and 3, and two loxP sites positioned between exons 2–3 and 4–5 of the *Usp21* locus. The vector was introduced into 129/Sv-derived ES cells, and G418-resistant clones were screened by PCR and Southern blot. Correctly targeted ES clones were injected into C57BL/6 blastocysts to generate chimeric mice. Germline-transmitting chimeras were crossed with C57BL/6 mice, and the neo cassette was removed by breeding with Flp recombinase-expressing mice. The floxed allele retained loxP sites flanking exons 3 and 4. Subsequent crossing with Cre recombinase-expressing mice excised these exons, introducing a frameshift and premature stop codon in exon 5, thereby generating constitutive *Usp21* KO mice. Genotypes were verified by PCR.

For experiments, 10–11 week old *Usp21* KO and WT littermate mice were used. Sample size was determined by animal availability and prior experience; no formal power calculation was performed. Group allocation was based solely on genotype. All mice were housed under identical conditions on a normal diet with a 12 h light/12 h dark cycle.

Investigators were aware of group assignments during animal handling, blood collection, and tissue harvesting. Biochemical assays and RNA sequencing were performed using standardized protocols without blinding. The primary outcome measure was the serum lipid profile, specifically T-CHOL, and FFA levels.

All animals were anesthetized to minimize distress, and no adverse events were observed. Sacrifice was performed at predetermined time points. All procedures were approved by the Institutional Animal Care and Use Committee (approval number: 1204160979; approval date: 5 April 2012).

### 4.2. DNA Electrophoresis

The RNA was isolated from the WT and *Usp21* KO mice and amplified using *Usp21*-specific primers by RT-qPCR. RNA was extracted using the RNeasy Plus kit (Qiagen, Hilden, Germany). The *Usp21* KO was confirmed with agarose gel electrophoresis. *mGapdh* was used as a reference gene. The primers are listed in Table S1.

### 4.3. RNA-Sequencing and Enrichment Analysis

RNA-seq was carried out using Illumina MiSeq (Illumina, San Diego, CA, USA) according to the manufacturer’s protocol. The RNA was extracted from the littermates WT and *Usp21* KO mice using the ISOGEN II reagent (NIPPON GENE, Toyama, Japan). The genes that were differentially regulated as a result of *Usp21* KO were analyzed using the Partek^®^ Genomics Suite^®^ (Partek, St. Louis, MO, USA). All the RNA-seq data can be found online in the NCBI GEO submission (GSE304655).

### 4.4. RT-qPCR

The total RNA was extracted using the ISOGEN II reagent (NIPPON GENE, Toyama, Japan). cDNA was synthesized using 0.5 μg total RNA as a template with oligo (dT) primer (Life Technologies, Carlsbad, CA, USA), random hexamers (Takara Bio, Kusatsu, Shiga, Japan), and M-MuLV reverse transcriptase (New England Biolabs, Ipswich, MA, USA). RT-qPCR was performed with an ABI PRISM 7900 HT (Applied Biosystems, Foster City, CA, USA) with SYBR green as a reporter, cDNA as a template, and gene-specific primers. The target gene expression levels were standardized to *Gapdh* expression levels. All the primers designed for the experiment are listed in Table S1.

### 4.5. Mice Serum Analysis

Serum samples were collected from 10–11-week-old littermate WT mice (total *n* = 21), *Usp21* KO mice (total *n* = 28), and fed with ND. Body weights were measured prior to sacrifice. 0.5–1.0 mL of blood was taken from the inferior vena cava. The collected blood was allowed to coagulate by keeping it at room temperature for 30 min to 2 h. The coagulated blood is then centrifuged at 3000 rpm for 10–30 min at room temperature. The supernatant separated as a result of centrifugation is the serum which is then cryopreserved to analyze the levels of TG, FFA, T-CHOL, AST, ALT, HDL-C, and LDL-C. TG, FFA, T-CHOL, AST, and ALT were measured using enzymatic colorimetric assay kits (FUJIFILM Wako Pure Chemical Corporation, Osaka, Japan). HDL-C and LDL-C were determined using direct enzymatic assays (Cholestest N HDL and Cholestest LDL; SEKISUI MEDICAL Co., Tokyo, Japan), a widely used method in clinical and experimental studies that does not require precipitation or ultracentrifugation. All mouse data are summarized in Table S2.

### 4.6. Liver Specimen Collection

10–11 weeks-old WT (*n* = 5) and *Usp21* KO (*n* = 5) littermate mice were sacrificed, and liver specimens were collected. Liver and spleen weights were measured, and the tissues were preserved in 4% formalin for subsequent staining.

### 4.7. HE Staining

Liver tissue specimens collected from 10–11 week old WT (*n* = 5), and *Usp21* KO (*n* = 5) littermate mice were paraffin-embedded, sectioned at 5 μm using a Leica HM325 microtome, and mounted onto glass slides. The slides were subsequently deparaffinized, stained with HE, and mounted. Finally, the prepared slides were observed under a microscope.

### 4.8. SNP Selection and Genotyping

All the individual samples are from participants who attended an annual health check-up. This annual check-up program is conducted by the local government and directed by the Ministry of Health, Labor, and Welfare in Japan. This study was approved by the Ethics Committee of Nagasaki University Graduate School of Biomedical Sciences (project registration number 14051404). Written consent forms were available in Japanese to ensure a comprehensive understanding of the study objectives, and informed consent was provided by the participants, Table S3. A 100 kb sequence upstream and downstream of *USP21* was identified from the NCBI database GENBANK (GRCh37.p9) and input to GENETYX-MAC software version 12.0 (GENETYX, Tokyo, Japan) to identify the restriction enzyme sites. All the SNPs within the restriction enzyme site around the *USP21* region were identified. A total of 22 SNPs were selected. A total of 67 patient samples were collected, of which 41 had lower concentrations of LDL-C (<50 mg/dL), and the remaining 26 displayed higher LDL-C levels (>160 mg/dL). All the patient’s cDNA was amplified by PCR with primers specific for each SNP. The primers for all 22 SNP are mentioned in Table S4. The PCR was performed using the GoTaq DNA polymerase (Promega Cat.No.M3008, Madison, WI, USA). The PCR master mix included 5xGoTaq buffer 2 μL, 2.5 mM dNTP 0.8 μL, 10μM forward primer 0.5 μL, 10 μM reverse primer 0.5 μL, Go Taq enzyme 0.05 μL, cDNA (about 100 ng/μL) 0.1 μL and distilled water to make up the total reaction volume to 10 μL. The fragments were amplified at 95 °C for 1 min for the melting step, followed by 35 cycles of amplification (95 °C for 1 min, 55 °C 1 min, 72 °C 30 s) and terminal extension at 72 °C for 5 min. Next, the PCR products were cut with specific restriction enzymes (PCR product 5 μL, 100X BSA 0.1 μL, 10X enzyme buffer 1 μL, restriction enzyme 0.3 μL and distilled water to make the volume up to 10 μL). The samples were incubated for 1 h at 37 °C and 3 μL of the restriction enzyme treated sample was run on 3% agarose gel electrophoresis at 170 V for 30 min. For one of the SNPs (rs2301286), sequencing was carried out to confirm the results or restriction enzyme cut. as the gel electrophoresis results were not clear. Sequencing was performed using the Big Dye^TM^ Terminator v3.1 (Thermo Fisher Scientific Inc, Waltham, MA, USA) according to manufactural protocol and analyzed with ABI PRISM 3130xl genetic analyzer (Applied Biosystems, Foster City, CA, USA). The characteristics of the SNPs and the restriction enzymes used are mentioned in Table S5. The frequency of the SNP alleles in patients is mentioned in Table S6.

### 4.9. Statistical Analysis

Data are shown as mean ± SD. Student’s *t*-test was used to determine the statistical significance for the target genes subjected to RT-qPCR, and to determine the statistical significance between the mice genotypes and blood test parameters. The chi-squared test was used to determine the statistical significance between the phenotypes and SNPs. *p* < 0.05 was considered significant for all the test data. All the statistical analyses were performed using JMP student edition 18 software (SAS Institute, Cary, NC, USA).

## Data Availability

The data supporting this study’s findings are available in the methods and Supplementary Materials of this article or in GEO. All the RNA-seq data can be found online in the NCBI GEO submission (GSE304655).

## Supplementary Materials

The following supporting information can be downloaded at: https://www.mdpi.com/article/10.3390/ijms26199727/s1.

## Abbreviations

ALT: alanine transaminase; ANOVA, analysis of variance; *Ajuba*, ajuba LIM protein; *Apcs*, amyloid P component, serum; *Acly*, ATP citrate lyase; *Acaca*, acetyl-Coenzyme A carboxylase alpha; *Acacb*, acetyl-Coenzyme A carboxylase beta; ASCVD, atherosclerotic cardiovascular diseases; AST, aspartate aminotransferase; *Apob*, apolipoprotein B; *ApoE*, apolipoprotein E; *Apoa1*, apolipoprotein A-1; BP, Biological phase; BPH, Biological Phase; BR, Biological regulation; BW, body weight; *Ccl2*, C-C motif chemokine ligand 2; *Ccr7*, C-C motif chemokine receptor 7; CP, Cellular process; *Cyp2a5*, cytochrome P450, family 2, subfamily a, polypeptide 5; D, Detoxification; DP, Developmental process; ex, excision; *Faap100*, Fanconi anemia core complex associated protein 100; *Fabp7*, fatty acid binding protein 7; FABPs, fatty acid binding proteins; *Fasn*, fatty acid synthase; *FCER1G*, Fc fragment of IgE receptor Ig; FFA, free fatty acids; fl, floxed; FRT, flippase recognition target sites; *Gapdh*, glyceraldehyde-3-phosphate dehydrogenase; G, Growth; *Gnat1*, G protein subunit alpha transducin 1; GO, gene ontology; *Hspa1b*, heat shock protein 1B; *Hspb1*, heat shock protein 1; HE, heterozygous; H&E, hematoxylin and eosin; *HDL-C*, high-density lipoprotein cholesterol; HP, Homeostatic process; HSV-tk, herpes simplex virus- thymidine kinase; INTER, Biological process involved in interspecies interaction between organisms; INTRA, Biological process involved in intraspecies interaction between organisms; ISP, Immune system process; KO, knockout; *Kit*, receptor tyrosine kinase; *Lpin1*, lipin 1; LW, liver weight; LDL, low-density lipoprotein; *LDL-C*, low-density lipoprotein cholesterol; *LDLR*, low density lipoprotein receptor; L, Localization; LO, Locomotion; MOP, Multicellular organismal process; MP, Metabolic process; *Mttp*, microsomal triglyceride transfer protein; neo, neomycin resistance gene; ND, normal diet; *Nlrc5*, NLR family CARD domain containing 5; *Nqo1*, NAD(P)H dehydrogenase, quinone 1; P, Pigmentation; *Ptch1*, patched 1; *Ppargc1a*; peroxisome proliferator activated receptor gamma coactivator 1 alpha *Pcsk9*, proprotein convertase subtilisin/kexin type 9; R, Reproduction; REP, Reproductive process; RP, Rhythmic process; RS, Response to stimulus; *Rxrg*, retinoid X receptor gamma; *Serpinh1*, serine (or cysteine) peptidase inhibitor, clade H, member 1, SNPs, single-nucleotide polymorphisms; SW, spleen weight; T-CHOL, total cholesterol; TG, triglycerides; TAM, tamoxifen; *Tlr12*, Toll-like receptor 12; ubH2A, ubiquitylated H2A; *Usp21*, ubiquitin specific peptidase 21; UTR, untranslated region; VP, Viral process; WT, wild-type.
