# Supplementary material for: Usp21 Knockout Causes Abnormal Lipid Metabolism in Mouse and Its Polymorphism Correlates with Hypercholesterolemia in Outpatients
Source: Int J Mol Sci. 2025 Oct 6;26(19):9727. doi: 10.3390/ijms26199727 (PMC12525000; doi:10.3390/ijms26199727)
Supplement: Supplementary file 1 [file ijms-26-09727-s001.zip › ijms-3838463-supplementary.pdf]

Supplementary Figure 1 Sailakshmi et al

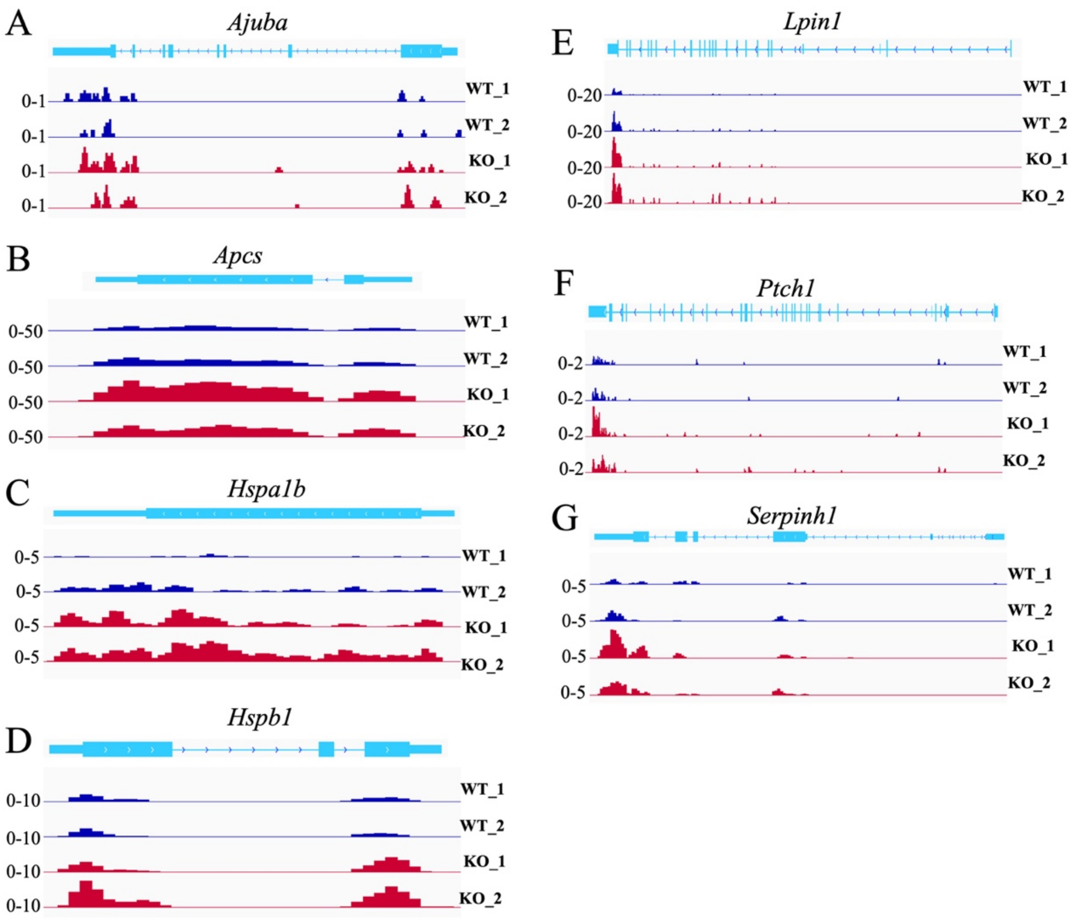

Supplementary Figure 1: (A-G) Chromosome view of up-regulated genes. (A) *Ajuba*, (B) *Apcs*, (C) *Hspa1b*, (D) *Hspb1*, (E) *Lpin1*, (F) *Ptch1*, *and* (G) *Serpinh1* in *Usp21* KO mice in comparison with WT mice.

Supplementary Figure 2 Sailakshmi et al

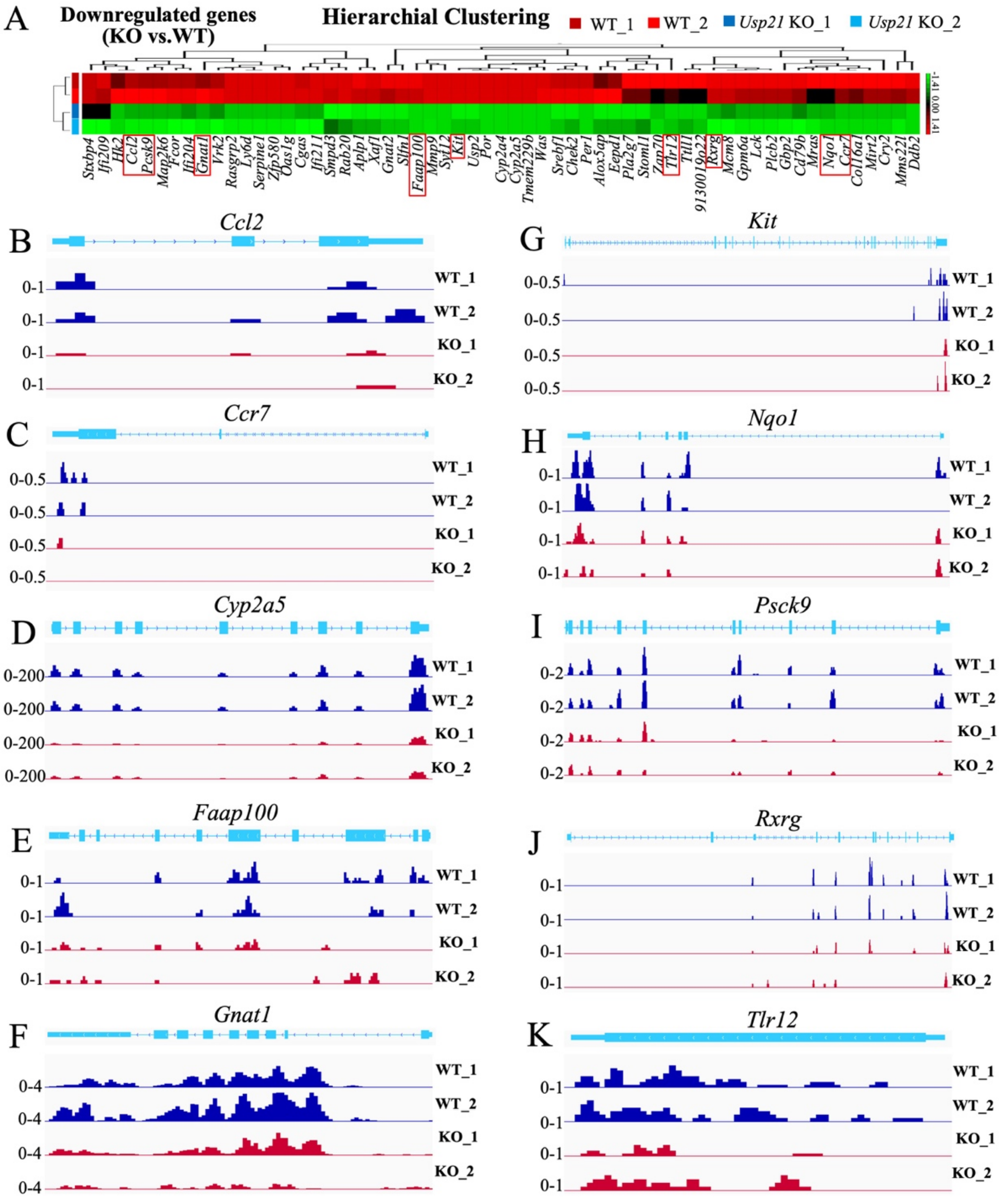

Supplementary Figure 2: (A) Heatmap of downregulated genes highlighting the top 10 genes in *Usp21* KO mice in comparison with WT mice. (B-K) Chromosome view of down-regulated genes. (B) *Ccl2*, (C) *Ccr7*, (D) *Cyp2a5*, (E) *Faap100*, (F) *Gnat1*, (G) *Kit*, (H) *Nqo1*, (I) *Psck9*, (J) *Rxrg*, (K) *Tlr12* in *Usp21* KO mice in comparison with WT mice.

Supplementary Figure 3 Sailakshmi et al

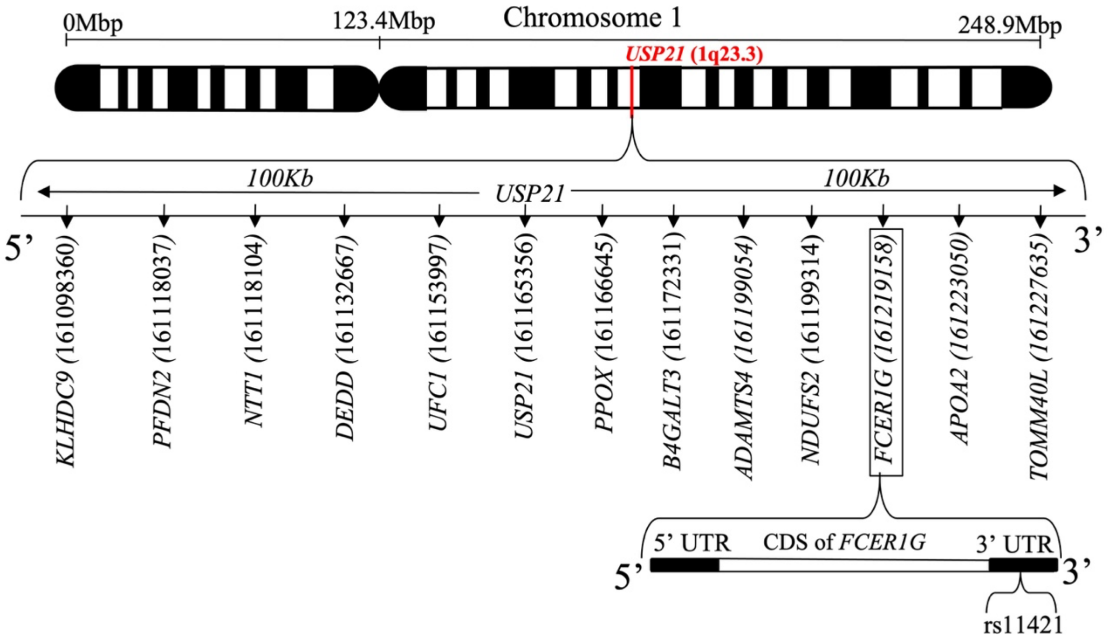

Supplementary Figure 3: The SNP rs11421 is situated in the 3′ UTR of the neighboring *FCER1G* gene, 53.8 kb away from the *USP21* gene.

Supplementary table 1		
**Gene Name**	**Direction**	**Primer**
Fabp7	Forward	5’-GCTGACTAGGCGGTTAAGG-3’
	Reverse	5’-GCCACCTTCCTGACTGATAATC-3’
Gapdh	Forward	5'- AGCCTCGTCCCGTAGACAA-3'
	Reverse	5'- ATGAAGGGGTCGTTGATGGC-3'
Nlrc5	Forward	5’-GCTGTCCGGGAATCTCCTTGG-3’
	Reverse	5’-GGACGCAGGATCCCTGGACC-3’
Ppargc1a	Forward	5’ -GTGTGTCAGAGTGGATTGG-3’
	Reverse	5’- GAAGATCTGGGCAAAGAGGCTG-3’
Usp21	Forward	5’ -ACTGGGCCTAGACCTAATTAATCC-3’
	Reverse	5’- GGTCATGAGTAGTAAGCCCATCAC-3’

Supplementary table 2

Number	Weeks	Genotype	TG (mg/dL)	FFA(μ/EQ/L)	T-CHOL (mg/dL)	AST (IU/L)	ALT (IU/L)	LDL-C (mg/dL)	HDL-C (mg/dL)	Liver weight (g)	Spleen weight (g)	BW (g)	Diet
1	11	KO	32	823	112	62	35	NA	NA	NA	NA	22.75	Normal diet
2	11	KO	25	624	118	56	45	NA	NA	NA	NA	23.76	Normal diet
3	11	KO	22	832	87	62	29	NA	NA	NA	NA	22.64	Normal diet
4	11	WT	28	767	81	77	67	NA	NA	NA	NA	22.65	Normal diet
5	11	WT	48	758	83	56	40	NA	NA	NA	NA	26.11	Normal diet
6	10	KO	53	762	107	46	25	NA	NA	NA	NA	24.18	Normal diet
7	10	WT	50	614	82	48	26	NA	NA	NA	NA	25.36	Normal diet
8	10	KO	48	690	112	62	28	NA	NA	NA	NA	25.8	Normal diet
9	10	KO	31	782	115	55	27	NA	NA	NA	NA	20.45	Normal diet
10	10	WT	22	628	72	60	15	NA	NA	NA	NA	20.85	Normal diet
11	10	KO	23	795	128	40	12	NA	NA	NA	NA	21.39	Normal diet
12	10	WT	11	654	70	44	13	NA	NA	NA	NA	15.9	Normal diet
13	10	KO	15	823	128	71	20	NA	NA	NA	NA	19.75	Normal diet
14	11	KO	32	884	125	44	28	NA	NA	NA	NA	20.64	Normal diet
15	11	KO	22	783	118	48	30	NA	NA	NA	NA	21.04	Normal diet
16	11	KO	33	768	122	51	28	NA	NA	NA	NA	20.19	Normal diet
17	10	KO	18	705	75	61	27	NA	NA	NA	NA	25.38	Normal diet
18	10	KO	24	700	111	47	24	NA	NA	NA	NA	21.38	Normal diet
19	10	WT	38	633	84	42	18	NA	NA	NA	NA	23.74	Normal diet
20	10	WT	51	536	76	41	19	NA	NA	NA	NA	23.61	Normal diet
21	10	KO	44	606	100	54	18	NA	NA	NA	NA	22.98	Normal diet
22	10	WT	22	603	89	44	23	NA	NA	NA	NA	25.39	Normal diet
23	10	KO	48	779	116	47	24	NA	NA	NA	NA	23.42	Normal diet
24	10	WT	24	521	69	63	25	NA	NA	NA	NA	18.38	Normal diet
25	10	KO	14	629	100	52	24	NA	NA	NA	NA	16.64	Normal diet
26	10	KO	15	827	104	52	22	NA	NA	NA	NA	18.01	Normal diet
27	10	WT	25	563	80	39	17	NA	NA	NA	NA	19.51	Normal diet
28	10	KO	35	773	93	55	28	NA	NA	NA	NA	20.41	Normal diet
29	10	WT	47	738	76	50	22	NA	NA	NA	NA	26.68	Normal diet
30	10	KO	71	638	121	47	33	NA	NA	NA	NA	25.15	Normal diet
31	10	KO	13	722	84	74	25	NA	NA	NA	NA	22.1	Normal diet
32	10	WT	16	564	56	60	16	NA	NA	NA	NA	19.97	Normal diet
33	10	WT	11	559	62	52	23	NA	NA	NA	NA	14.84	Normal diet
34	10	KO	31	746	98	44	18	NA	NA	NA	NA	18.45	Normal diet
35	10	WT	35	585	84	57	29	NA	NA	NA	NA	24.65	Normal diet
36	10	WT	13	358	61	54	17	NA	NA	NA	NA	23.54	Normal diet
37	10	KO	24	631	90	49	26	NA	NA	NA	NA	18.16	Normal diet
38	10	WT	20	418	76	47	27	NA	NA	NA	NA	23.03	Normal diet
39	10	KO	20	431	122	58	32	NA	NA	NA	NA	24.29	Normal diet
40	11	WT	NA	403	81	NA	NA	3	57	1.3393	0.0696	29	Normal diet
41	11	WT	NA	838	78	NA	NA	6	54	1.2746	0.0662	29	Normal diet
42	11	WT	NA	664	66	NA	NA	4	45	1.4595	0.1053	32	Normal diet
43	11	WT	NA	843	73	NA	NA	4	47	1.3795	0.0877	30	Normal diet
44	11	WT	NA	650	73	NA	NA	4	46	1.2236	0.0776	29	Normal diet
45	11	KO	NA	773	79	NA	NA	5	50	1.032	0.0765	26	Normal diet
46	11	KO	NA	407	75	NA	NA	7	47	0.9407	0.0825	26	Normal diet
47	11	KO	NA	742	125	NA	NA	8	93	1.3763	0.0971	28	Normal diet
48	11	KO	NA	1387	126	NA	NA	6	92	1.7989	0.107	31	Normal diet
49	11	KO	NA	2014	110	NA	NA	10	66	1.3577	0.1045	28	Normal diet

Supplementary table 3

Sample number	Age	Gender	Height (cm)	Weight (kg)	Body fat percentage (%)	Waist circumference (cms)	BMI	LDL Cholesterol (mg/dL)
1	74	F	149	53	29.1	78	23.87279852	58
2	75	F	144.6	44.6	28	72	21.3303643	58
3	72	M	162.9	43.3	12.7	70	16.31720342	31
4	24	F	162.3	48.2	21.3	62	18.29826861	56
5	67	M	164	51	N/A	72	18.96192742	48
6	70	F	152	56	N/A	88	24.23822715	56
7	72	F	144	50	N/A	84	24.11265432	51
8	64	F	142	53	N/A	87.5	26.28446737	43
9	72	F	153	46	21.8	76	19.65056175	42
10	67	M	168	57	22.5	80	20.19557823	46
11	73	F	146	35	17.1	66	16.41959092	48
12	46	F	169	56	21.2	74.5	19.60715661	57
13	54	F	153	44	19.7	64	18.7961895	51
14	70	M	N/A	N/A	N/A	N/A	N/A	52
15	69	M	160	64	20.7	89	25	43
16	66	M	163	55	18.3	76	20.70081674	39
17	79	F	143	36	14.4	61.5	17.60477285	37
18	63	F	143	62	39.3	99	30.31933102	46
19	72	M	160	51	13.3	77.5	19.921875	47
20	60	M	159.2	52.8	N/A	75	20.83280725	53
21	56	M	168	78	N/A	102.5	27.63605442	57
22	83	F	133.4	40.6	31.1	71.9	22.81467962	52
23	64	F	151.2	54	31.7	87	23.62055933	45
24	73	F	150.6	40.6	18.8	77.5	17.90095043	54
25	77	M	162.8	70.6	22.2	93.7	26.63764949	49
26	65	F	153	49	24.8	76.8	20.93212012	49
27	49	M	165	67	18.2	84.2	24.6097337	38
28	73	M	159	69	20.5	96.5	27.29322416	47
29	68	M	165.7	53.7	14.7	75	19.55821738	49
30	74	M	162	54.2	15.6	78.6	20.65233958	49
31	79	F	146.9	51.3	25	92.3	23.77244074	53
32	78	F	144.2	48.7	20.6	93	23.42062284	40
33	55	M	163.2	49.7	11.6	73.4	18.66019079	37
34	75	M	162.9	65.5	14.4	88.4	24.68306753	55
35	59	M	163.9	67.5	25.1	86.8	25.12730236	47
36	49	F	152.3	45.1	21.3	67.3	19.44360256	56
37	63	M	167.3	60.5	12.3	74.3	21.61541075	55
38	65	M	164.1	60.3	16.1	76	22.39237456	53
39	63	F	153.5	45.7	20	73.5	19.39543125	54
40	73	F	149.3	60.3	N/A	97.6	27.05189523	57
41	76	M	162.5	65.3	29.1	91.2	24.72899408	55
42	82	F	142.7	43.4	N/A	78	21.31286251	221
43	66	F	153.8	60.5	N/A	83	25.57659365	223
44	66	F	153.9	57.4	30.5	75	24.23453286	231
45	63	F	153.6	61.7	32.3	81	26.15186903	255
46	58	F	152	56.5	29.6	73	24.45463989	217
47	64	F	152.8	60.2	33.4	84	25.78396974	229
48	74	F	152.8	58.7	36.2	86.5	25.14151202	225
49	75	F	149.8	50.8	27.1	81	22.63810581	217
50	59	M	168	59	N/A	74	20.90419501	241
51	57	F	159	71	N/A	96	28.08433211	248
52	72	F	142	49	N/A	87	24.30073398	249
53	57	F	151	59	N/A	89	25.87605807	219
54	57	M	163	67	N/A	84	25.21735858	223
55	61	F	155	61	32.3	85	25.39021852	222
56	65	F	147	51	27.4	74.2	23.60127725	217
57	52	F	156	54	N/A	86.5	22.18934911	258
58	60	F	152	64	N/A	91.6	27.70083102	251
59	52	F	148.5	44.1	29.4	79.5	19.99795939	218
60	79	F	135.3	59.6	N/A	100	32.55747131	237
61	59	F	149	51	29.8	83.2	22.9719382	233
62	74	F	148.4	56	31.6	88.5	25.42846971	227
63	68	F	151.9	59.2	33.8	89.2	25.65701683	219
64	65	F	146.1	42.9	26.3	68.9	20.09818596	222
65	83	F	143.1	47	26.3	77.7	22.95189722	247
66	44	F	157.9	44.9	18.8	75.3	18.00868829	236
67	74	M	162.1	71	20.6	N/A	27.02043392	236

Supplementary table 4		
**Ref_SNPs**	**Direction**	**Primer**
rs17384360	Forward	5’-TTCCTTACTGCAACCTCCGC-3’
	Reverse	5’TCCTTCACCTTATGTAGAGTGATAAG-3’
rs4617422	Forward	5’-TTCATCCTCCTGCACACCAC-3’
	Reverse	5’AGGTGACAGCAGACTATTTATGAGTC3’
rs3841330	Forward	5’-ACAGGCATCACGTCATCCAAC-3’
	Reverse	5’-AGCAGGCAGTCACATTTTCC-3’
rs3820097	Forward	5’TACTTGGAGAGAATTTCTGGATTGG3’
	Reverse	5’-AGGCGGATGAGGGCGACTAC-3’
rs72480265	Forward	5’-ATCTCCAGCATCTAGTATCC-3’
	Reverse	5’-TGACTCTGAACTGAGGAGCC-3’
rs4656287	Forward	5’-ATACCAGTCGCTAGCCCTAG-3’
	Reverse	5’-AACTACAGTGATGCAAAAATGATAG3’
rs4523530	Forward	5’-TCCTCTGCTGCCATAATGAAG-3’
	Reverse	5’-ATTGGATAACTCTGGATAATG-3’
rs12041364	Forward	5’-TGAGATTGTGCCACCGCACTAC-3’
	Reverse	5’-TGCCTTCAAGTCAACTGTGTC-3’
rs11265560	Forward	5’-TGCCTGGAACATATTAGGAAC-3’
	Reverse	5’AGTGTGTAGTCCCTGCTACTCGGAGG3’
rs12031437	Forward	5’-AGTTCCCATCCTATGAGTCT-3’
	Reverse	5’-TAACAAAGACCAAACCTCAG-3’
rs2301286	Forward	5’AACGCTAACTCCAGCATTAAGCGC-3’
	Reverse	N/A
rs2301287	Forward	5’-TAGATGGATCCTGGCCCTCTG-3’
	Reverse	5’-ACTAGGACCACCTGGCGACAAG-3’
rs11265562	Forward	5’-TCCATCCACTGACTCTGATTC-3’
	Reverse	5’-AAATTATGACCCATAAACTG-3’
rs11265563	Forward	5’-TGGTCTCAAACTGCTAACCTC-3’
	Reverse	5’-TTAGATACAGAAGGAGACAG-3’
rs7512012	Forward	5’-TTGGTAAATCAAACGAGACC-3’
	Reverse	5’-TGCTGGCTAGATGTTATATG-3’
rs1136224	Forward	5’-TGGAGCCTGTTCCTCACTGG-3’
	Reverse	5’-TTAGTACTTGCACAGAAAGC-3’
rs11421	Forward	5’TCATATTCTTCTTTGGCTTCTGGTTC3’
	Reverse	5’-AGTCCATGGCAGTTTTATTGG-3’
rs3813627	Forward	5’AAGGGTTAAATACAGAAAGCAGCTG-3’
	Reverse	5’-TGGAGGTCTACATCTCTCGAAG-3’
rs9725457	Forward	5’-TGCCTTGACCTCTCAAAGTG-3’
	Reverse	5’-AATAATAATAAGGTGCCCTTGTCGG-3’
rs4656293	Forward	5’-AGGATGGTCTCGATCTCCTG-3’
	Reverse	5’-TCAGTATGCTTTTTCTGTATCAACC3’
rs6671288	Forward	5’-TGGCCCACAGCATAGTAACTATTTAG3’
	Reverse	5’-AAGTCCTGTCTCCTAGAGTCTAGC-3’
rs2501866	Forward	5’-TCTATTTGTAGGCACAGTCTCATC-3’
	Reverse	5’-ATTGAGTGCCAGGTCCTGTTTC-3’

Supplementary table 5								
**Annotation of SNP**	**Ref_SNP**	**Reference genome assembly**	**Chromosome**	**MAF**	**Ref_SNP alleles**	**Restriction enzyme**	**Chromosome**	**Chromosome position**
1	rs17384360	GRCh37.p5	1	G=0.218/477	G/T	MseI	1	161030806
2	rs4617422	GRCh37.p5	1	A=0.287/628	A/G	AvrII	1	161036472
3	rs3841330	GRCh37.p5	1	A=0.276/603	-/AT	NlaIII	1	161044838:161044839
4	rs3820097	GRCh37.p5	1	G=0.143/313	A/G	RsaI	1	161049358
5	rs72480265	GRCh37.p5	1	A=0.101/220	A/G	MspI	1	161057428
6	rs4656287	GRCh37.p5	1	C=0.101/221	C/T	MseI	1	161064008
7	rs4523530	GRCh37.p5	1	T=0.390/852	G/T	NlaIII	1	161084773
8	rs12041364	GRCh37.p5	1	A=0.301/658	A/G	TaqI	1	161110347
9	rs11265560	GRCh37.p5	1	C=0.263/574	C/T	AluI	1	161118134
10	rs12031437	GRCh37.p5	1	G=0.376/821	C/G	HaeIII	1	161126975
11	rs2301286	GRCh37.p5	1	A=0.383/837	A/C	NlaIII	1	161136224
12	rs2301287	GRCh37.p5	1	G=0.376/821	C/G	HpaII/MspI	1	161136843
13	rs11265562	GRCh37.p5	1	A=0.269/587	A/T	MseI	1	161149863
14	rs11265563	GRCh37.p5	1	A=0.266/580	A/G	BstUI/HinP1I	1	161151844
15	rs7512012	GRCh37.p5	1	G=0.308/673	A/G	NaeI	1	161162173
16	rs1136224	GRCh37.p5	1	G=0.169/370	A/G	NlaIII	1	161184097
17	rs11421	GRCh37.p5	1	C=0.212/463	C/T	XspI	1	161188936
18	rs3813627	GRCh37.p5	1	T=0.338/737	G/T	BfaI	1	161195148
19	rs9725457	GRCh37.p5	1	A=0.344/751	A/G	NlaIII	1	161201574
20	rs4656293	GRCh37.p5	1	T=0.184/402	C/T	AluI	1	161218094
21	rs6671288	GRCh37.p5	1	G=0.136/296	C/G	ApaI	1	161219555
22	rs2501866	GRCh37.p5	1	C=0.274/599	A/G(C/T)	HaeIII	1	161229684

Supplementary table 6						
	**High LDL cholesterol patients**			**Low LDL cholesterol patients**		
**Ref_SNPs**	**Homozygous minor allele**	**Heterozygous allele**	**Homozygous major allele**	**Homozygous minor allele**	**Heterozygous allele**	**Homozygous major allele**
rs17384360	2	13	11	1	14	26
rs4617422	2	12	12	7	20	14
rs3841330	1	11	14	1	18	22
rs3820097	1	10	15	1	17	23
rs72480265	1	7	18	1	9	31
rs4656287	1	7	18	1	9	31
rs4523530	11	13	2	14	20	7
rs12041364	9	14	3	12	20	9
rs11265560	8	14	4	12	21	8
rs12031437	11	14	1	14	20	7
rs2301286	11	14	1	14	20	7
rs2301287	11	14	1	14	20	7
rs11265562	9	11	6	12	17	12
rs11265563	8	14	4	13	19	9
rs7512012	0	1	25	0	3	38
rs1136224	1	9	16	4	19	18
rs11421	10	12	4	3	19	19
rs3813627	1	13	12	10	21	10
rs9725457	11	13	2	9	21	11
rs4656293	5	11	10	1	19	21
rs6671288	0	2	24	0	4	37
rs2501866	4	12	10	5	13	23
